# Diagnosis of hepatitis C virus infection in pregnant women in the healthcare system in Poland: Is it worth the effort?

**DOI:** 10.1097/MD.0000000000004331

**Published:** 2016-07-29

**Authors:** Bożena Walewska-Zielecka, Urszula Religioni, Grzegorz Juszczyk, Aleksandra Czerw, Zbigniew Wawrzyniak, Piotr Soszyński

**Affiliations:** a Medical University of Warsaw; b Warsaw School of Economics; c Warsaw University of Technology, Warsaw, Poland.

**Keywords:** hepatitis C virus, obligatory screening, pregnant women

## Abstract

The hepatitis C virus (HCV) is globally recognized as a serious public health concern. Current statistics indicate that approximately 2% of people worldwide and 1.9% of people in Poland suffer from HCV infection.

This study was conducted to assess the anti-HCV seroprevalence in pregnant women in Poland and subsequently provide recommendations on the rationale for obligatory screening.

A total of 42,274 women participated in our study, of which 16,130 were pregnant. We were granted access to their health data stored in the form of electronic medical records kept by the network of outpatient clinics throughout Poland.

The lowest rate of positive anti-HCV test results was found in women ages 25 to 34 (0.73%); however, younger and older age groups had similar rates (15–24 = 0.86%; 35–44 = 0.84%). Additional analysis of data from the period between 2011 and 2014 revealed a downward trend in the proportion of positive anti-HCV tests among pregnant women (mean positive anti-HCV = −0.001 × year + 1.9451; *R*^2^ = 0.7274). Regardless of the gradual increase in the number of female patients undergoing screening between 2004 and 2015, there has been a constant decrease in the rate of positive cases. The rate of pregnant women potentially infected with HCV was twice as lower than that in a control group of women undergoing tests for other medical circumstances: 0.76% vs 1.67% (*P* < 0.0001).

Analysis of real-world data of female patients in Poland provides evidence that screening based on an individual's medical history and behavioral risk factors in clinical circumstances would be more effective than obligatory testing of all pregnant women.

## 1 Introduction

Hepatitis C virus (HCV) infection is considered to be a serious public health concern by the World Health Organization (WHO) and one of the major public health priorities. The most common available marker of HCV infection consists of the antibodies to HCV (anti-HCV). The prevalence of HCV infection ranges from 1.2% to 3.8% in different regions of the world. The highest prevalence of HCV is noted in Central Asia (3.8%), East Asia (3.7%), and North Africa/Middle East (3.6%).^[1,2]^

The antenatal prevalence of infection varies from 1.0% to 2.5% in Europe to more than 10.0% in some sub-Saharan countries, and mother-to-child HCV transmission risk ranges from 3.0% to 10.0%. Vertical transmission of HCV is considered the primary route of HCV infection in children.^[3,4]^ The presence of maternal HCV RNA at the time of delivery and maternal co-infection with HCV and human immunodeficiency virus (HIV) are the most important factors for the increased risk of perinatal HCV transmission.^[5]^

So far, the research on HCV infection in Poland has mostly focused on the prevalence of HCV infection in specific risk groups, possible diagnostic methods, or descriptions of clinical cases. However, available data may not accurately reflect the actual prevalence of HCV infection due to different methods of data collection. Therefore, our study is based upon a wide range of patient data obtained from medical records of countrywide outpatient clinics. It is important to point out that in 2012, the Polish Government introduced the obligatory anti-HCV test for all pregnant women in Poland in order to reduce the risk of vertical infection to their children.

## 2 Objective

The objective of the study is to estimate the anti-HCV seroprevalence in pregnant women in Poland and formulate recommendations on the rationale of such a screening accordingly.

## 3 Patient and methods

**Figure 1:**
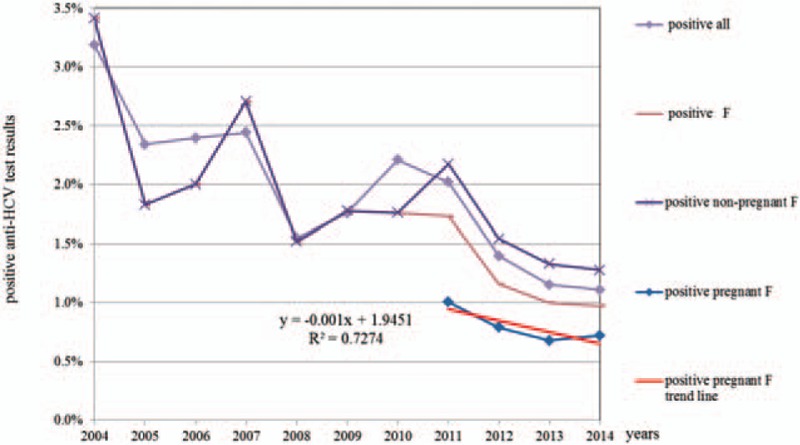
Percentage of positive anti-hepatitis C virus test results within the examined female population from 2004 to 2014.

Data were obtained in February 2015 from electronic medical records of a large countrywide outpatient managed care clinic's network operating mainly in large cities. De-identified, aggregate data used for this study were publicly accessible and therefore ethical approval was not required. The data contained medical information of patients who had been tested for anti-HCV at least once. Only the test latest result was included in the study pool, which finally comprised 42,274 women in the period of 2004 to 2014 (Table 1, Fig. 1). The studied group included a population representative of the working age, 15 to 64 years. The working age population was defined according to the WHO definition. The average age of the women was 33.4 years with a standard deviation of 7.9 (Table 2). The results of the pregnant women were compared to the corresponding age groups of nonpregnant women and with all women in the study sample. Data were analyzed using the data analysis software system STATISTICA version 12 (StatSoft, Inc., Warsaw, Poland; www.statsoft.com) to calculate prevalence estimates. The independent-sample *t* test was used for normally distributed variables, and the nonparametric Mann–Whitney *U* test was used for not normally distributed parameters. Significance level was set at *P* < 0.05. Using linear regression analysis, the trend of the number of positive women as a function of time (years) was calculated and the *R*^2^ value evaluated the goodness of fit of the regression (Fig. 1, Tables 1 and 2).

**Table 1:**
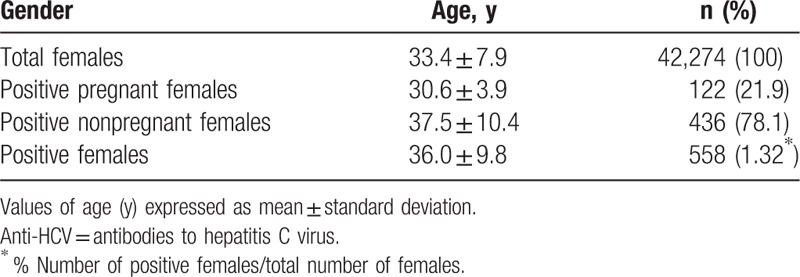
Age and percentage of positive anti-HCV females.

**Table 2:**
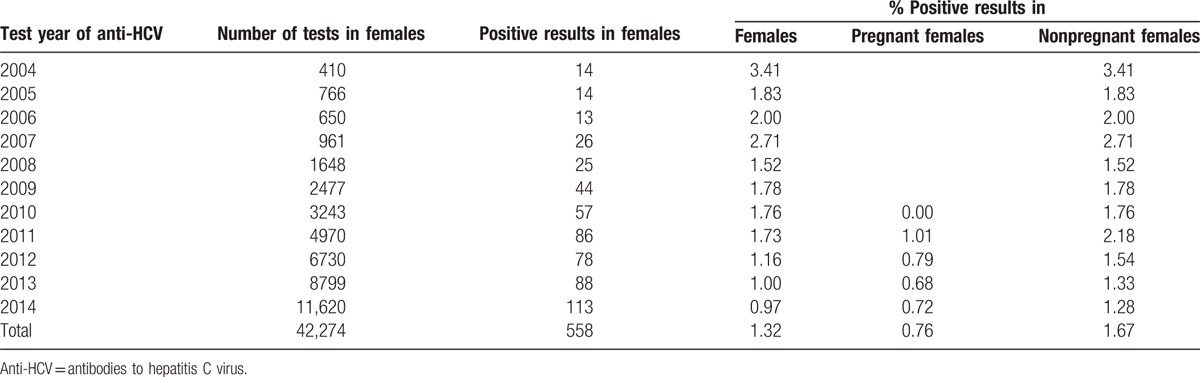
Results of anti-HCV tests for the study population in the period of 2004 to 2014.

## 4 Results

Table 3 shows the numbers and percentages of positive women per age group. In the group of tested women, the percentage of those with positive anti-HCV test results was similar across age groups; it was the lowest among women ages 25 to 34 (0.73%) and 35 to 44 (0.84%), which was similar to that of the youngest group, 15 to 24 years (0.86%). An analysis of the data of pregnant women for the years 2011 to 2014 shows a downward trend of the proportion of positive anti-HCV results (linear trend for number of positive anti-HCV pregnant females = −0.001 × year + 1.9451; *R*^2^ = 0.7274; *P* < 0.001; Fig. 1). With the systematic growth in the number of tested women in the years 2004 to 2015, a decrease in the percentage of positive anti-HCV test results was observed. The prevalence of potential HCV infection was almost 2 times lower in pregnant women (0.76%) than in the other cases, which were patient requests or doctor referrals (1.67%; *P* < 0.0001).

**Table 3:**
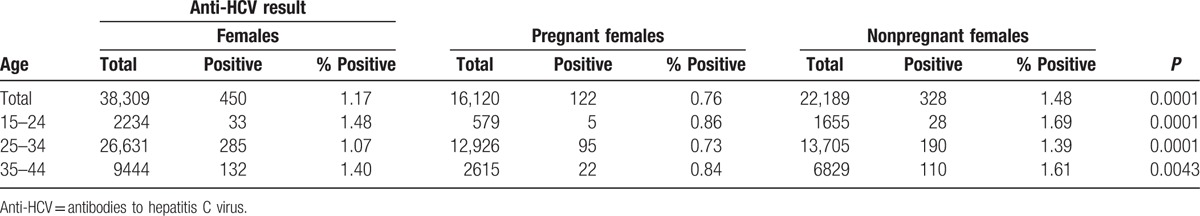
Results of anti-HCV tests for the different age groups in the study population.

For the pregnant women of all 3 age groups (15–24, 25–34, and 35–44 years) the percentage of positive test results was 0.86% or lower, which is still far less than the lowest value of 1.39% for the group of 25- to 34-year-old nonpregnant women (*P* < 0.0001).

## 5 Discussion

WHO data show that the prevalence of HCV infection in European countries is estimated at 2.4% for Central Europe, 2.9% for Eastern Europe, and 2.4% for Western Europe. Over half of the cases of HCV infection in Europe occur in the age group of 25- to 44-year-olds and are more common in men (64.4%) than women (35.6%). The available data also demonstrate that the number of cases of HCV infection in Western Europe is decreasing, while it is increasing in Eastern Europe.^[2,6,7]^

In the United States, the HCV infection prevalence is 1.6% (more common in men with 2.1% than in women with 1.2%); however, a higher prevalence (75% of all cases) was observed in people born between 1945 and 1965.^[8]^ For this reason, the US Center for Disease Control and Prevention (CDC) in 2012 recommended screening in the form of a disposable diagnostic test available for all individuals born in these years. According to new data from the CDC, the number of persons with chronic HCV infection was about 0.5 million fewer than previously estimated. However, this estimate might have been influenced by the increasing number of deaths due to HCV complications, which have been observed in recent years in the United States. The high cost of HCV infection treatment, estimated at $80,000 per treatment course, is often not covered by insurance companies, which is a barrier for the majority of infected patients.^[9]^

The results of the present study show, however, that the prevalence of HCV infection in pregnant women has been lower than estimated (at approximately 2.0%),^[10,11]^ and the proportion of the screened population is substantial (all pregnant women in Poland estimated annually at around 300,000). The costs for both public and private payers who have to provide reimbursement of anti-HCV screening could be spent on other important prenatal activities, especially considering the fact that infected mothers are highly unlikely to infect their children.

On the basis of the analysis of risk groups, it can be estimated that each year, HCV is spread to over 5 million infants by their infected mothers worldwide. This disease remains usually undetected until liver symptoms appear in adulthood. This problem is particularly serious in countries where the prevalence of HCV is high and mothers are co-infected with HIV.^[1]^ Among mothers without HIV infection, HCV transmission risk is estimated at 4% to 8%, while among mothers with HIV infection it increases to 17% to 25%.^[4]^ Research in 2008 in a group of 544 pregnant women showed a prevalence of HCV infection in 2.02% of tested women.^[12]^ According to the authors’ own research on 16,130 pregnant women, the prevalence of HCV in these female patients was only 0.73%, and this figure is close to the European average (1%).^[6]^

Although selective antenatal HCV screening has been the most common measure for prevention of HCV in infants, its cost-effectiveness has not been proven. Another insufficiently proven recommendation is cesarean delivery for HCV-positive women co-infected with HIV.^[13]^

## 6 Conclusion

According to our research, we recommend that screening for HCV should be done mainly in individuals over 45 years old. Examining healthy and young people should not be carried out as a regular screening test. However, such tests may be recommended for individuals who have been exposed to risk factors, as established through a thorough interview by a physician. Obligatory screening in pregnancy according to the results on a large group of women in Poland has proven less effective that screening in other clinical circumstances based on individual medical history and behavioral risk factors. Thus, as a recommendation, healthcare providers and payers should consider developing internal guidelines and standards for anti-HCV screening with definitions of suspected cases, instead of relying on such general characteristic like pregnancy.

The results of evaluating anti-HCV prevalence in the real-world daily patient flow may be helpful for public health authorities and policy makers. These results may be used in estimating the potential costs of treatment for HCV infection, as well as treating complications resulting from the infection.

## Acknowledgments

The authors would like to express their gratitude to the Board of Medicover Ltd. in Poland for granting free-of-charge access to the anonymized set of data from patients’ electronic medical records.
